# To sleep or not to sleep: the ecology of sleep in artificial organisms

**DOI:** 10.1186/1472-6785-8-10

**Published:** 2008-05-14

**Authors:** Alberto Acerbi, Patrick McNamara, Charles L Nunn

**Affiliations:** 1 Max Planck Institute for Evolutionary Anthropology, Deutscher Platz 6, 04103 Leipzig, Germany; 2 Department of Neurology, Boston VA Medical Centre and Boston University School of Medicine, Boston, MA 02130, USA; 3 Department of Integrative Biology, University of California, Berkeley, CA 94720, USA

## Abstract

**Background:**

All animals thus far studied sleep, but little is known about the ecological factors that generate differences in sleep characteristics across species, such as total sleep duration or division of sleep into multiple bouts across the 24-hour period (i.e., monophasic or polyphasic sleep activity). Here we address these questions using an evolutionary agent-based model. The model is spatially explicit, with food and sleep sites distributed in two clusters on the landscape. Agents acquire food and sleep energy based on an internal circadian clock coded by 24 traits (one for each hour of the day) that correspond to "genes" that evolve by means of a genetic algorithm. These traits can assume three different values that specify the agents' behavior: sleep (or search for a sleep site), eat (or search for a food site), or flexibly decide action based on relative levels of sleep energy and food energy. Individuals with higher fitness scores leave more offspring in the next generation of the simulation, and the model can therefore be used to identify evolutionarily adaptive circadian clock parameters under different ecological conditions.

**Results:**

We systematically varied input parameters related to the number of food and sleep sites, the degree to which food and sleep sites overlap, and the rate at which food patches were depleted. Our results reveal that: (1) the increased costs of traveling between more spatially separated food and sleep clusters select for monophasic sleep, (2) more rapid food patch depletion reduces sleep times, and (3) agents spend more time attempting to acquire the "rarer" resource, that is, the average time spent sleeping is positively correlated with the number of food patches and negatively correlated with the number of sleep patches. "Flexible" genes, in general, do not appear to be advantageous, though their arrangements in the agents' genome show characteristic patterns that suggest that selection acts on their distribution.

**Conclusion:**

Collectively, the output suggests that ecological factors can have striking effects on sleep patterns. Moreover, our results demonstrate that a simple model can produce clear and sensible patterns, thus allowing it to be used to investigate a wide range of questions concerning the ecology of sleep. Quantitative data presently are unavailable to test the model predictions directly, but patterns are consistent with comparative evidence from different species, and the model can be used to target ecological factors to investigate in future research.

## Background

Sleep is an evolutionary puzzle. Unlike activities such as mating, foraging and seeking shelter, the functional benefits of sleep are unclear, and the costs of sleep appear to be substantial. Most animals thus far studied exhibit some kind of sleep, including fruitflies [1], jellyfish [2], birds [3], reptiles [4] and mammals [5]. Animals exhibit incredible variation in the duration of sleep. In mammals, for example, the armadillo (*Chaetophractus villous*) can sleep up to 20 hours per day [6], while a giraffe (*Giraffa camelopardalis*) sleeps for only two hours per day [7,8]. Similarly, animals exhibit variation in the timing of sleep throughout the day. One obvious aspect of this involves activity pattern, i.e. whether animals sleep during the day, night, or some mixture of day and night (e.g. cathemerality). Another aspect of sleep timing involves the number of sleep bouts per day. Some species, such as cats, sleep in multiple bouts, making them polyphasic; other species, such as many primates, tend to be monophasic, with a single sleep bout per day [9,10].

Researchers have for some time investigated potential evolutionary functions of sleep [11-13]. Most efforts aimed at understanding the evolution of sleep have focused on factors that influence the duration of sleep, with emphasis on possible physiological benefits obtained from sleep. For example, many authors have proposed that sleep durations vary with restorative properties of sleep, while others have noted that sleep durations are linked with the consolidation of memories (see chapters in [14]). Thus, we might predict that species with greater memory needs, such as food-storing birds, would devote more of their daily time budgets to sleep. Other proposed benefits of sleep include bodily repair, energy conservation, and release of hormones that govern growth and reproduction [5,15]. It seems likely that sleep provides some benefits, given that sleep deprivation leads to a sleep-rebound effect, whereby an animal must sleep for a longer time to make up the sleep deficit [16].

In comparison to proposed physiological benefits, less attention has been given to the ecological factors that influence the time available for sleep in different species. For example, foraging constraints might limit the time available to sleep [12]. Thus, many small mammals, such as shrews, have limited time available to sleep because they live on a metabolic "knife-edge," with a need to eat as often as every two hours [17,18]. Similarly, an animal that eats a food resource that is distributed in small patches is likely to spend more time traveling among food patches, giving less time for sleep. If sleeping animals are more vulnerable to predation, then one might expect that predation risk could also act as a selective pressure to reduce the amount of time available for sleep [12,19,20]. Other ecological factors that could potentially limit the time available for sleep include reproductive competition, the distance between sleep and food patches, and time involved in food processing.

The role of some of these ecological factors in structuring the amount of time animals spend sleeping has been investigated by comparing sleep 'quotas' or durations across different species. For example, comparative studies have revealed a negative relationship between predation risk and sleep duration [12,19,21], that polyphasic sleepers sleep longer than monophasic sleepers [9,22], and that polyphasic sleep is associated with energetic constraints of small body mass [22]22. Along similar lines, Lima *et al*. [20] investigated the links between predation and sleep quotas, focusing in particular on the ways that predation risk might vary during REM versus NREM sleep and the consequences of this for the evolution of sleep. Behaviorally, we also know that animals exhibit sleep related behaviors that are expressed in an ecological and social context. Many primates, for example, sleep in particular trees or on cliff ledges [23], while a variety of rodents sleep in underground burrows, many of which can represent a substantial investment of energy to build and maintain [24]. Thus, sleep sites do not occur randomly in the habitat and can require significant travel to reach.

In short, ecological influences on sleep likely act as significant constraints on species' sleep quotas. Within these constraints, we might then expect that the physiological benefits of sleep will accrue at different rates, as demanded by the organism and altered through natural selection [21]. Assuming, for example, that memories are consolidated during sleep, an ape that has high demands for memory consolidation could achieve these benefits through either a longer duration of sleep, or through an increased rate of memory consolidation during the period of sleep. Understanding the nature of ecological constraints is therefore a crucial first step in understanding inter-specific variation in sleep quotas.

In this paper, we develop an evolutionary agent based model to investigate how ecological constraints might impact sleep patterns. *Agent based *modeling aims to understand global dynamics of ecological systems by simulating local interactions between individuals, or agents [25]. In the case of *evolutionary *agent based models, a certain number of agents' characteristics are encoded in free parameters (an artificial genome) and optimized using techniques of evolutionary computation, such as genetic algorithms [26]. This method is useful for identifying the range of parameter values that is selected by the evolutionary algorithm, depending on variation in environmental factors that are controlled by the experimenter. This, in turn, can help to identify aspects to further analyze in empirical studies, being in particular useful for phenomena that are challenging to observe in real animals (or difficult to reproduce in controlled experimental conditions). Despite the importance of sleep in the behavioral repertoire of animals, and the significant role that ecological factors are likely to play in shaping sleep patterns, remarkably few researchers have attempted to model sleep using artificial organisms (but see [27,28]; another approach is presented in [29]).

We investigated three ecological factors that are expected to have a major influence on sleep patterns: the spatial distribution of sleep and foraging sites; the food depletion rate, i.e. the time an animal can spend feeding until a food patch is depleted; and the number of food and sleep sites in the environment. We focus on three primary aspects of sleep as dependent variables: the total duration of sleep per 24-hours, the phasing of sleep-wake cycles, measured as the number of sleep bouts (monophasic versus polyphasic sleep) per 24 hours, and whether animals sleep flexibly (i.e., when short of sleep relative to food) or inflexibly (i.e., specific blocks of time per day).

## Methods

We follow the standard protocol identified by Grimm *et al*. [30] to describe our model. This protocol, known as ODD, involves seven elements that form the structure of our Methods description. We then add a section (Simulation Experiments) in which we describe how experiments are performed (number of repetitions, initial values of state variables, etc.)

### Purpose

The purpose of our model is to investigate how sleep characteristics, such as the total duration of sleep, the phasing of sleep-wake cycles (monophasic versus polyphasic sleep, measured as number of sleep bouts), and whether agents sleep flexibly or inflexibly, are impacted by ecological factors. In particular, we focus on three ecological factors: (1) the spatial distribution of sleep and foraging sites, specifically regarding the spatial overlap of food and sleep sites; (2) the food depletion rate, i.e. the time spent feeding until resources are depleted, thus forcing an individual to locate a new feeding site; and (3) the number of food and sleep sites in the environment.

### State variables and scales

Agents are characterized by several variables. First, each agent has a location in the landscape. Second, agents in the simulation have two energy levels that are each adjusted positively when eating and sleeping occur. One concerns "sleep energy" and the other concerns "food energy." Third, agents have a circadian rhythm that is determined by 24 "genes" (one for each hour of the day – see below) that are subjected to evolution by means of a genetic algorithm. Each gene has one of three possible states that determine the actions of individuals: sleep or search for a sleep site, eat or search for a food site, or flexibly decide action based on relative levels of sleep energy and food energy (described below).

**Figure 1:**
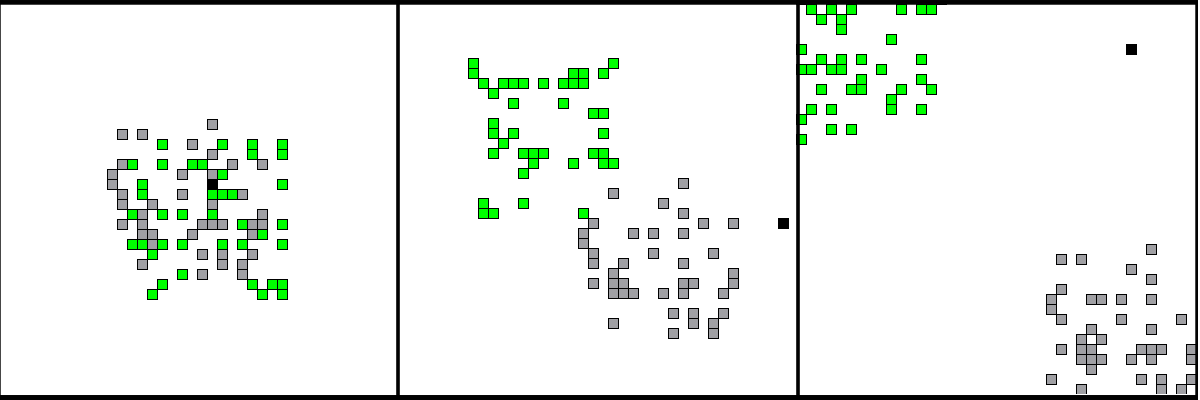
**Snapshots of the lattice with different *interDistance *values**. The distribution of food (green) and sleep (gray) clusters vary with *interDistance *values of 0.0, 0.35 and 0.7, and *intraDistance *fixed at 0.2 (default for all simulations). The number of food and sleep patches was constant (40 sleep patches and 40 food patches). The black dot shows an agent.

The simulation runs on a square lattice of 40 × 40 cells with a hard boundary. Habitat types are mutually exclusive, with each cell in the habitat matrix identified as being either a sleep patch (i.e. a cell of the lattice in which agents can sleep), a food patch (i.e. a cell of the lattice in which agents can eat), or nothing. The numbers of sleep and food patches and the degree to which these resources overlap can be varied, but sleep and food patches are always arranged in "clusters" of a fixed radius (*intraDistance*). We specify a distance parameter that determines the distance between the center of the sleep cluster and the center of the food cluster (*interDistance*). Figure 1 provides examples of *interDistance *values representing the range of parameters used here; *intraDistance *was fixed at 0.2 for all simulations.

The model runs in discrete time steps. We identify the following terms in this context: minute, hour, day, generation and simulation run. Agents operate on time steps of one minute and seek food or sleep at each time step based on their circadian rhythm. An hour is used as the unit in the circadian rhythm of an agent, with 24 genes – one for each hour of a day – that determine the action of the agent at any given time in the 24-hour cycle. A generation is considered to encompass the life of an agent and lasts for 7 days. One hundred agents are tested for each generation and each simulation run covers 100 generations.

### Process overview and scheduling

After initializing the landscape with food and sleep sites, the simulation proceeds, at each time step by inspecting the appropriate gene (for a given hour) on an agent's genome. Depending on what the gene specifies, the agent moves or stays in the cell in which it is located. The state of the environment is updated after the agent's movement. Agents live in separate landscapes and thus do not interact with one another. At the end of the 7-day generation, reproduction takes place, as described below.

### Design concepts

#### Fitness

**Figure 2:**
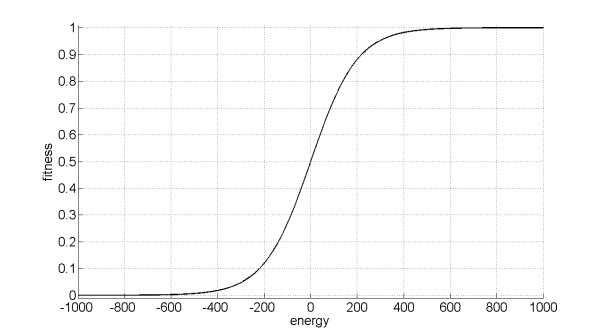
**Energy/fitness transformation**. The curve shows how the energy (x-axis) is transformed into the actual fitness of the agents (y-axis). See also Equation 1.

Sleep and food energy are translated into fitness measures at every time step. Specifically, relationships between energy and fitness are nonlinear and calculated explicitly, with fitness bounded between 0.0 and +1.0 using a sigmoid function:

(1) $$f=\frac{1}{1+\exp^{-\frac{1}{100}\cdot energy}}$$

where *f *= fitness (see figure 2). The overall fitness of agents is represented by the average of the two fitness measures.

#### Sensing

Agents can only sense the cell in which they are currently located and thus can not make decisions about movement to neighboring cells based on their content. This helps to simplify the model and, as considered in the Discussion, we have no reason to expect that a more complicated model of sensing or use of mental maps would qualitatively change the conclusions drawn from our model. Agents also have a rough knowledge of their energy level (i.e., if energies differ by more than 10 percent of the maximum fitness values), which allows them to switch between activities when in a "flexible" state.

#### Observation

We examined output from the last generation of each experiment, focusing on the average number of sleep bouts per 24-hour cycle, the percentage of monophasic sleepers in the population, the average number of sleep genes in individual genomes, the average number of flexible genes in individual genomes, and the average total number of minutes of sleep in the last day (i.e., based on actual behavior rather than the genes coding behavior, as sleep sites may not be found each time that a gene specifies sleep). We assume that a sleep bout ends when the agent is awake for more than 30 consecutive minutes. Agents that exhibit only one sleep bout during a 24-hour period (including a single sleep bout that spans the end of one day and beginning of the next) are considered monophasic sleepers. For one experimental condition (see section *Input parameters*) we also recorded the structure of the genome of the best evolved agent in each run.

In all cases, we excluded runs in which the average fitness of the population remained under a value of 0.9, as this indicates that the agents were unable to solve the task given to them, due to the difficulty of the task or possibly due to stochastic effects. That, in turn, ensures that the population evolved to a stable equilibrium point.

### Initialization

The habitat matrix is formed by placing a given number of food and sleep patches on the lattice within the constraints of the *interDistance *and *intraDistance *parameters. (see figure 1) The agent is randomly located on a cell in the matrix. Each agent is tested in an independent lattice with the same set of initializing parameters, but with stochastic differences in the distribution of food sites and sleep sites. Energy levels for the agent are set to zero, which corresponds to a fitness of 0.5 (see figure 2). The starting conditions for the genome are randomly selected from among the three states, resulting in an expected distribution of eight sleep genes, eight feeding genes, and eight flexible genes (although actual initial numbers were determined stochastically and thus could vary among individuals).

### Input

The model does not include any environmental data. See "simulation experiments" for details on the ecological parameters that were varied.

### Submodels

#### Movement

Agents move throughout the lattice in a correlated random walk. Specifically, at each time step, agents modify their current direction of travel with a random value between -15 degrees and +15 degrees; thus, major changes in direction do not occur on the order of a single time step. Agents remain in the same cell during a time step only when they locate a food or sleep patch corresponding to their genetic coding for a given hour of the day (sleep or eat). Agents can move to any of the surrounding cells on flat sides and corners of their current cell, thus giving a total of eight possible moves per time step. If the gene specifies sleep, the agent moves until it locates a sleep site. It then remains at that sleep site until the genome specifies another action. If the gene specifies food, the agent moves until it locates a food site. It then remains until either the genome specifies a new action or until the food patch is depleted. If the gene specifies flexible action, the agent's activity is determined by its food and sleep energy levels, with behaviors driven by differences in energy. If the difference between the two energies is less than 10 percent of the maximum fitness value (see the *Fitness *subsection above), agents continue doing what they were last doing. In all other cases, agents perform the activity with the lower energy level.

#### Energy dynamics

The energy that agents can obtain when they are placed in a food patch declines linearly. Agents acquire sleep energy upon finding a sleep site. In the same way, food energy is acquired when an agent locates a food site. When not sleeping or eating, both sleep and food energies decrease. The ratio between the rate at which energy is gained on a sleep or food patch and the energy that is lost when not in a sleep or food patch was held constant after pilot experiments determined an appropriate level (3:1) that assured that the populations were able to find adaptive solutions in most of the experimental conditions.

#### Food depletion

Once the energy for a food patch is depleted, the patch disappears, and another patch is generated at a random, unfilled location in the lattice, subject to the *interDistance *and *intraDistance *constraints. The time until a food patch disappears is measured by the *food depletion rate *parameter. A lower depletion rate is equivalent to more resources in a food patch; higher rates mean that agents will deplete the patch more rapidly, and must therefore locate another food site to continue feeding. Unlike food patches, sleep sites do not disappear; thus, agents do not need to move to a new sleep site to continue obtaining benefits of sleep.

#### Reproduction

At the end of each generation, the 20 agents with the highest total fitness levels produce five offspring each; the remaining 80 individuals in each generation do not reproduce, thus maintaining a constant population size of 100 individuals per generation. Among the genomes that are passed to the next generation, mutation occurred at a rate of 5% per gene. When a mutation occurs, the current value of a gene changes to one of the other two values with equal probability.

### Simulation experiments

Table 1 provides details on the main parameters. We ran in total 5 experimental conditions defined by variation in: (1) the distance between the centers of the food cluster and the sleep cluster (*interDistance*), (2) the total number of sleep patches (keeping the number of food patches constant), (3) the total number of food patches (keeping the number of sleep patches constant), (4) the total number of sleep and food patches (keeping the two amounts equal to each other), and (5) the food depletion rate. Each experimental condition consisted in varying one of the parameters from the value *Min *to the value *Max *with an increment of *Step*, keeping all the other parameters constant. In initial runs of the simulation, we found that the parameter *interDistance *had a strong effect on our results. We therefore used three different constant values of *interDistance *for the experiments in which other parameters were varied (Table 1). To keep the size of food and sleep clusters constant across simulations, *interDistance *was constrained by edge limits of the matrix to be less than 0.7 (with *intraDistance *= 0.2); otherwise some sites would spill over the edge of the total spatial matrix.

**Table 1:** Parameters and their values used in the simulation experiments

**Parameter Name**	**Min**	**Max**	**Step**	**Constant**
Distance between sleep and food (*interDistance*)	0.0	0.7	0.01	0.0, 0.35, 0.7
Food depletion rate	10	240	1	60
Number of food patches	10	120	1	40
Number of sleep patches	10	120	1	40
Number of sleep and food patches	10	120	1	40

"Min" and "Max" represent the extreme ranges of values in focused tests, holding other traits constant. "Step" refers to the amount that values were incremented, while "Constant" gives the values used when one of the other parameters was varied.

In addition, to analyze the relative distribution of the three possible genes in the genome of the most successful agents, we ran a focused test consisting of 100 simulations with *interDistance *= 0.7 and all other parameters taking their "constant" values in Table 1. In order to realize this analysis, we first eliminated gene repetitions and then compared the frequency of three possible cases: E – F – E (a flexible phase between two eating phases), S – F – S (a flexible phase between two sleeping phases), and S – F – E or E – F – S (a flexible phase as a transition between a sleeping phase and an eating phase or vice versa). We then calculated an expected matrix using methods to control for the fact that sequences with adjacent identical values (e.g. S – S – E) were not possible in the observed sequence matrix [31]. Finally, we calculated the preferences of the three possible cases for each of the 100 evolved genomes, with preference = (observed – expected)/expected.

## Results

### The cost of travelling between spatially separated patches selects for monophasic sleep

**Figure 3:**
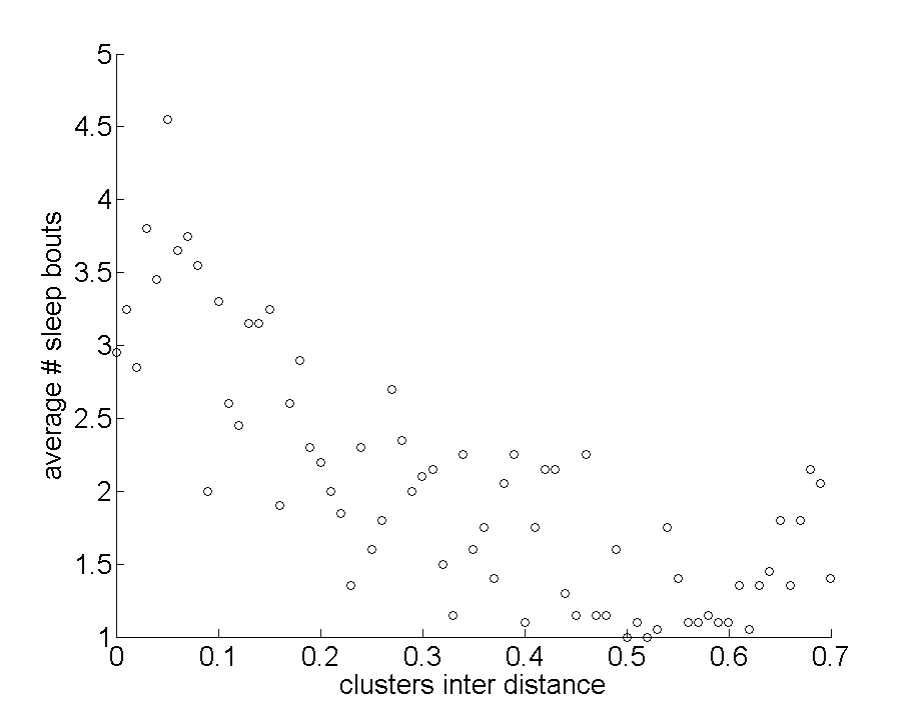
**Effects of *interDistance *on number of sleep bouts**. Average number of sleep bouts varying *interDistance*: *r*_*S *_= -0.76, *N *= 71, *P *< 0.001.

**Figure 4:**
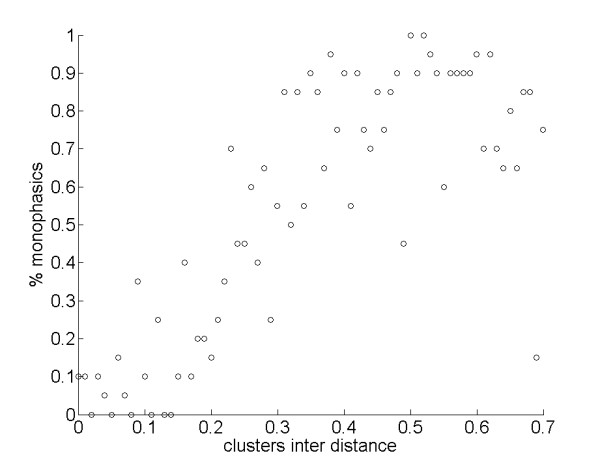
**Effects of *interDistance *on percentage of monophasic sleepers in the population**. Percentage of monophasic sleepers in the population varying *interDistance*: *r*_*S *_= 0.74, *N *= 71, *P *< 0.001.

The degree to which sleep and food patches overlapped had a substantial influence on whether agents slept polyphasically or monophasically. Varying the value of the parameter *interDistance*, which determines the degree of overlap between the food and sleep clusters (see Figure 2), we found that cluster overlap had a strong effect on the phasing of sleep-wake cycling over the 24-hour period. Specifically, reducing overlap in the distributions of food and sleep sites selected for fewer sleep bouts (Figure 3), and thus a tendency for monophasic sleeping behavior (Figure 4).

### Rapid food depletion reduces sleep time

**Figure 5:**
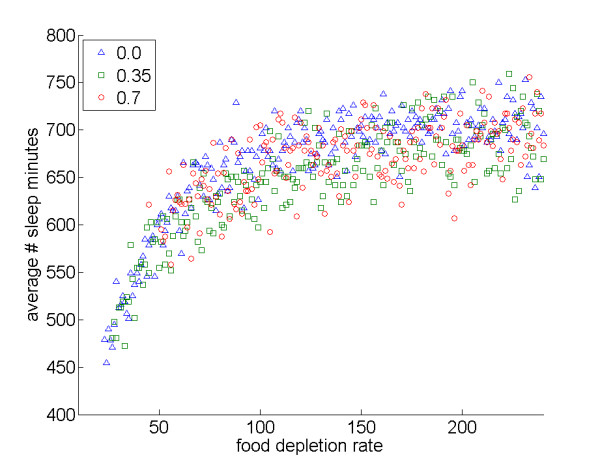
**Effects of food patch depletion rate on average sleep time**. Average number of sleep minutes varying food patch depletion rate (higher values represent slower rates of patch depletion): *interDistance *0.0: *r*_*S *_= 0.77, *N *= 218, *P *< 0.001; *interDistance *0.35: *r*_*S *_= 0.79, *N *= 214, *P *< 0.001; *interDistance *0.7: *r*_*S *_= 0.59, *N *= 190, *P *< 0.001.

Food depletion rates, measured as the time an agent spends feeding until a food patch is depleted, influenced sleeping patterns, with more rapid depletion negatively impacting sleep times in our simulations (Figure 5).

### Agents spend more time attempting to acquire the "rarer" resource

**Figure 6:**
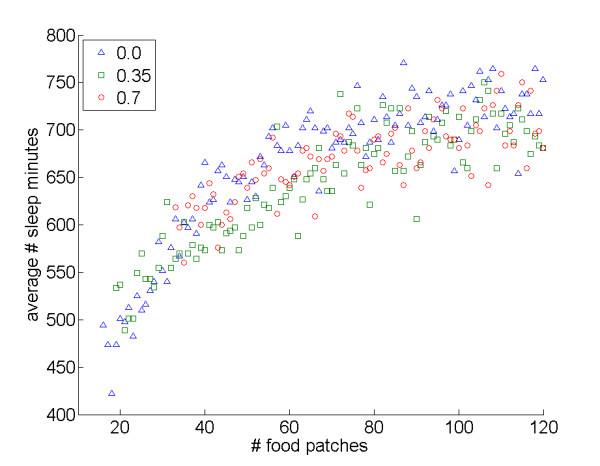
**Effects of the number of food patches on average sleep time**. Average number of sleep minutes varying the number of food patches (holding the sleep patches number constant): *interDistance *0.0: *r*_*S *_= 0.88, *N *= 105, *P *< 0.001; *interDistance *0.35: *r*_*S *_= 0.87, *N *= 102, *P *< 0.001; *interDistance *0.7: *r*_*S *_= 0.76, *N *= 88, *P *< 0.001.

**Figure 7:**
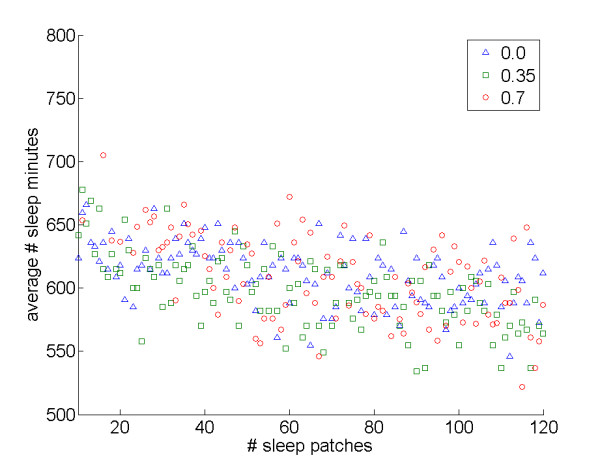
**Effects of the number of sleep patches on average sleep time**. Average number of sleep minutes varying the number of sleep patches (holding the food patches number constant): *interDistance *0.0: *r*_*S *_= -0.47, *N *= 111, *P *< 0.001; *interDistance *0.35: *r*_*S *_= -0.59, *N *= 111, *P *< 0.001; *interDistance *0.7: *r*_*S *_= -0.52, *N *= 99, *P *< 0.001.

The simulations further indicated that the number of food and sleep patches also can impact sleep patterns. When holding the number of sleep sites constant, an increase in the number of food sites tended to show a pattern of increased total sleep time (Figure 6); by comparison, when holding the number of food sites constant, an increase in the number of sleep sites reduced total sleep time (Figure 7). The results suggest that agents benefit from spending more time attempting to acquire the "rarer" resource, as might be expected if the benefits of the rare resource are more difficult to obtain.

**Figure 8:**
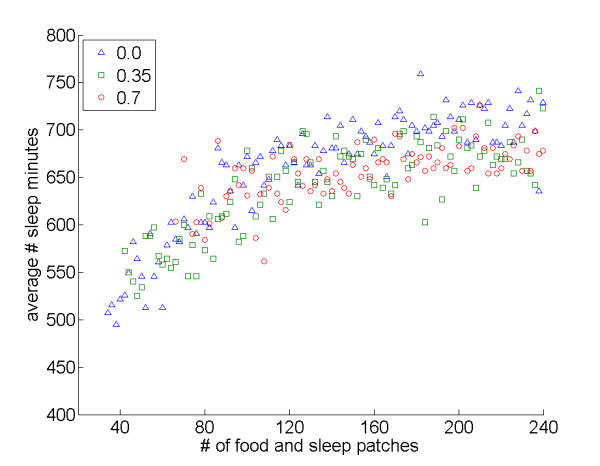
**Effects of the number of food and sleep patches on average sleep time**. Average number of sleep minutes when the number of food patches equals the number of sleep patches. Values on the x-axis are the sum of food and sleep patches (e.g., 80 refers to 40 food patches and 40 sleep patches): *interDistance *0.0: *r*_*S *_= 0.86, *N *= 104, *P *< 0.001; *interDistance *0.35: *r*_*S *_= 0.74, *N *= 100, *P *< 0.001; *interDistance *0.7: *r*_*S *_= 0.61, *N *= 84, *P *< 0.001.

Overall, however, an increase in both resource types tended to augment sleep time (Figure 8). This pattern can be explained by the fact that an increase in both resource types makes the overall task straightforward, so that agents can relatively easily spend more time sleeping (the same can be said for the time spent eating).

### The flexible strategy

We also considered whether a "flexible" strategy of sleep can evolve. We expected that flexibility would be favored, given that it would allow an agent to preferentially focus on the resource type that is most needed to increase fitness. Surprisingly, however, selection did not favor the spread of flexible genes. Specifically, the ending number of flexible genes tended to be similar or smaller than the initial expected value of eight flexible alleles (based on random assignment of 3 possible alleles to 24 different genes). When statistically significant effects were found for analyses of flexible genes, the actual effects were relatively weak in magnitude.

To better understand the role of the flexible genes we ran a focused test, consisting of 100 simulations with all the parameters values constant and *interDistance *= 0.7. In this test, the average number of flexible genes for the 100 fittest individuals in the last generation of each simulation was 6.67 ± 1.19, indicative of weak selection *against *flexibility.

**Figure 9:**
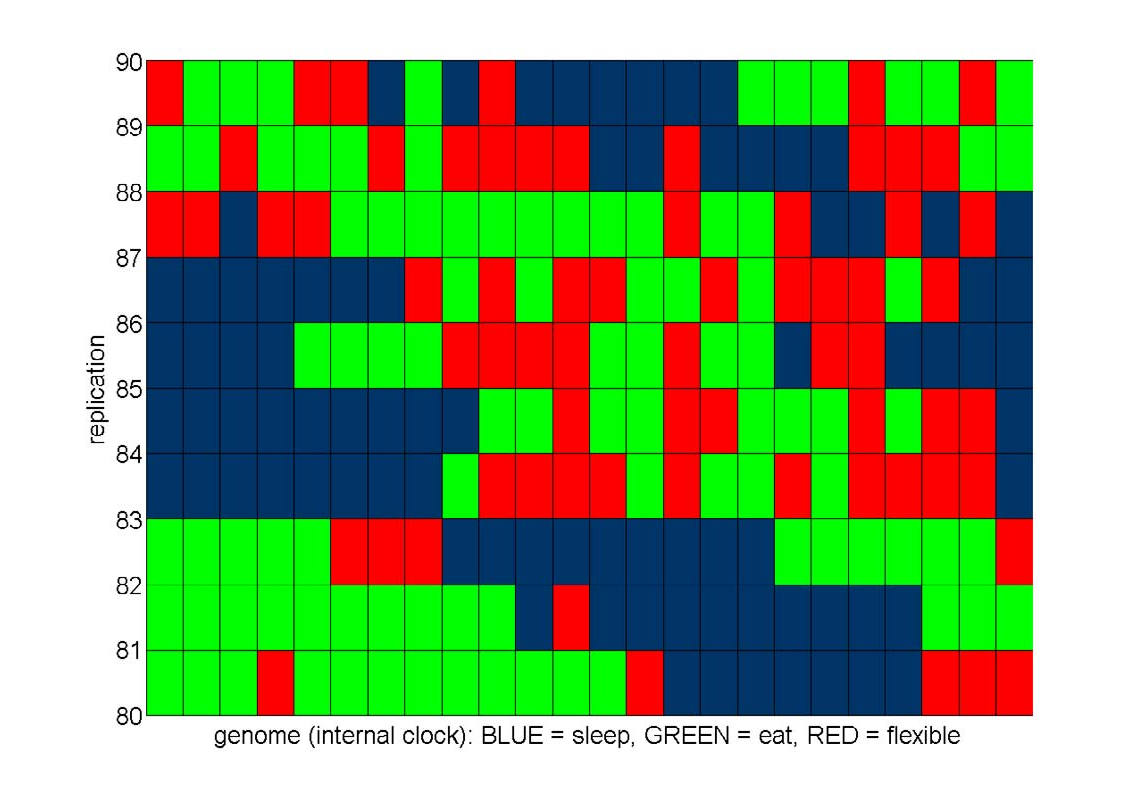
**The agents' internal clock**. A sample of 10 replicates (out of a total of 100) with all the  parameters values constant (*interDistance *= 0.7) showing the genome  (internal clock) of the best-evolved agents of the last generation.  Blue squares represent "sleep" genes, green squares "eat" genes, and  red squares "flexible" genes.

**Figure 10:**
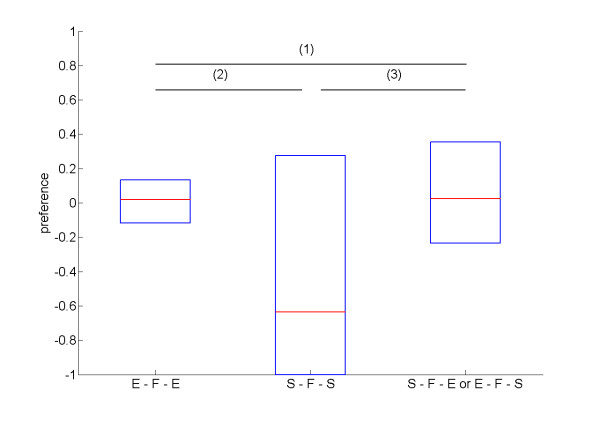
**Preferences for the flexible genes triplets**. E = eat (or search for a food site), F = act flexibly based on relative energy levels, and S = sleep (or search for sleep site). The boxes show median and percentiles for the three possible relative positioning of flexible genes, respectively, from left to right, E - F - E (a flexible phase between two eating phases), S - F - S (a flexible phase between two sleeping phases), and S - F - E or E - F - S (a flexible phase as a transition between a sleeping phase and an eating phase or vice versa). The mean rank for E - F - E is 2.08; 1.73 for S - F - S; and 2.19 for S - F - E or E - F - S combined together (Friedman test: χ^2 ^= 11.2, *df *= 2, *p *= 0.004). A post-hoc comparison confirms the significance for difference (2), between E-F-E and S-F-S (Wilcoxon test: *T*^+ ^= 2819.5, *N *= 90, *p *= 0.002) and difference (3), between S-F-E or E-F-S and S-F-S (Wilcoxon test: *T*^+ ^= 2854, *N *= 92, *p *= 0.005) but not for difference (1), between E-F-E and S-F-E or E-F-S (Wilcoxon test: *T*^+ ^= 2541, *N *= 98, *p *= 0.682).

Stronger effects were found concerning the positioning of flexible genes within the genome (see Figure 9). To further examine selection acting on the positioning of flexible genes in these 100 individuals, we analyzed their genomes in more detail. The analysis revealed that flexible genes were more commonly found at (1) the transition between sleeping and eating portions of the genome and (2) within blocks of eat genes rather than during sleeping phases, at least for monophasic sleepers (see Figure 10 for details).

## Discussion

Our simulation results revealed that ecological factors could strongly influence sleep characteristics. Four major conclusions arise from this work. First, the degree to which sleep and food sites overlap can greatly influence whether animals sleep polyphasically or monophasically: with less overlap, agents tended to sleep monophasically. This effect arises from the challenge of switching activities when food and sleep sites are more spatially separated, and is consistent with findings showing that monophasicity is expected in animals when the cost of changing between wakefulness and sleep is high [9].

Second, patch depletion rates influenced animal sleeping patterns, with more rapid depletion negatively impacting sleep times. As in the case of cluster overlap, this result involves the time costs of locating resources; in the case of food patch depletion, however, it reflects the costs of switching from one food site to another food site, rather than switching between food and sleep sites. Again, comparative evidence suggests that foraging constraints can have a great impact in reducing sleep time for real-world animals [12,21]. For example, animals with higher metabolic rates for their body mass spend less time asleep, as expected if they must spend more time foraging to meet their metabolic needs [21].

Third, the number of food and sleep sites impacted sleep patterns, with agents spending more time attempting to acquire the "rarer" resource, while an increase in both resource types tended to favor sleep. Some empirical research is also consistent with this finding. For example, sleep researchers who work with laboratory animals have noted that *ad libitum *diets – which should make sleep rare relative to food – are associated with increased sleep times when compared to restricted diets (see for example [32]).

Finally, we found that a flexible strategy is not strongly selected for or against by the evolutionary algorithm, but that selection acts on the relative positioning of flexible genes with respect to sleep and eat genes. In particular, flexible genes are more likely to be found during transitions between sleep phases and foraging phases, or within blocks of eat genes, rather than during sleeping phases. The greater occurrence of flexible genes at sleep-eat transitions suggests that agents can exploit flexibility to carry out a more fine-grained transition between the sleeping and the eating phases, giving the agent the ability to concentrate on a goal it has been unlucky in pursuing in the previous phase. The latter finding can be explained by the fact that, in contrast to sleep patches, food patches deplete over time. During the eating phase, the depletion of food patches forces the agents to move away from one patch and to resume searching for a new patch. The costs associated with switching activity while searching would likely be considerably lower than a switch in activity when agents are located in a sleep site. Thus, the transient nature of food patches may allow flexible genes to persist within blocks of eat genes.

This set of simulations focused on a few key parameters involving the distribution of food resources. As noted in the *Background *section, however, many other ecological factors could influence sleep patterns. For example, predation can greatly impact patterns of sleep, depending on whether safe sleep sites can be located [18]. When safe sleeping sites are unavailable, e.g. because an animal is too large or the habitat is open, we predict that a predator will have a strong negative impact on sleep times. Similarly, animals that forage visually will be able to locate food more efficiently in daylight, or might have to adjust the timing of their activity based on the activity of prey that are strictly diurnal or nocturnal. Thus, primates may find it more difficult to locate fruit or digestible leaves in nighttime conditions, while owls might hunt at night because it overlaps with the activity of their small-bodied rodent prey. These ecological constraints would be expected to strongly impact patterns of sleep in real organisms, especially with regard to the presence of monophasic sleep patterns. Environmental characteristics in our simulation were produced with few stochastic effects, especially regarding the number of food sites and the patch depletion rate. In the real world, the rate at which patches are depleted is likely to vary among sites, and this variability could impact sleep traits. Similarly, in a given simulation run, the number of food and sleep sites was held constant over the 7-day generation, whereas "seasonal" variation in these parameters could occur. Such variation could select for more flexible foraging strategies, thus modifying some of our conclusions. These factors could be included in future extensions of the model, with the prediction that flexible sleep patterns are more likely when stochasticity in initial conditions occurs, or when resources exhibit temporal variation over the life of the agents.

We also used a very simple movement strategy, with the agents in the model following a correlated random walk when searching for food or sleep sites. This means that switching from feeding to sleeping (or vice versa) entailed a random search for a new site, and this should make switching between resources more time consuming when food and sleep sites are more spatially separated. Indeed, we found that more simulations were excluded at high *interDistance *values, due to an average fitness lower than 0.9; thus, agents were unable to solve the task that we provided for them. Many real-world animals probably have a mental map that can guide their localization of new patches, and this could be particularly helpful when food and sleep sites show low spatial overlap. For example, hamadryas baboons probably do not randomly search the environment for their sleeping cliffs [33]; instead, they seem to know how to locate these critical resources more efficiently, perhaps through some form of mental map. While having a mental map would reduce the degree to which patch overlap influences monophasic sleep, we expect that the effects documented here will persist even with agents that have a mental map. As food and sleep sites become more spatially separated, this entails movement between patches; the mental map reduces the time required to move between different types of patches, but it does not eliminate the greater travel distance that is required, which is effectively a minimum fixed cost. Thus, a hamadryas baboon still has to invest the time to travel from food to sleep sites and this will be greater than in other species that sleep in closer proximity to food sites. Based on this reasoning, a mental map might alter some of the effects we found with *interDistance*, but it is unlikely to eliminate them.

We also assumed that agents are restricted to sleep only in particular habitats, designated here as sleep sites. This assumption is valid for many real-world species, but not for all of them. Some large-bodied African ungulates, for example, may be less constrained by spatial location than by social context; they probably obtain more benefits from reducing predation through close proximity with conspecifics than by finding safe refuge sites. Thus, elaborations of our model that are parameterized for particular species may require that social context be taken into account. Indeed, many mammalian species co-sleep throughout their life cycles. Co-sleeping in primates is associated with reduced predation rates, formation of social alliances and food-sharing, and feeding patterns [23]. Interactions among conspecifics will also increase food patch depletion rates, thus potentially increasing the effects of ecology on sleep patterns. As a related point, our model assumed that the agents did not interact. Future simulations could relax this constraint, as it is likely that both sleep times and sleep strategies (monophasic versus polyphasic) are influenced by the presence of conspecifics.

## Conclusion

Models such as ours can be used to identify traits to investigate in future field and comparative studies. Our results suggest in particular that three traits would be worthwhile to investigate. First, it would be interesting to study how the distribution of sleep sites influences the occurrence of monophasic sleep, with the prediction that monophasic sleep is more likely to be found in individuals or species in which the distribution of sleep and food sites overlap less. Second, patch depletion rate should have an important impact on the duration of sleep: when patch depletion is high, animals must locate new foraging patches, resulting in less time available for sleep. Finally, we predict that a greater availability of food patches will increase sleep times.

In conclusion, we showed that agent-based, computational approaches can be used to uncover the links between ecology and sleep. This model leaves out many realities of real organisms, and we do not intend this model to be a representation of any particular real organisms. Rather, this simplicity is necessary for developing an initial model of sleep ecology. Our results show that such a simple model can produce clear and sensible patterns, and some general patterns have already emerged that can be tested empirically. Finally, our results provide a first step towards developing the theoretical scaffolding to pursue additional theoretical research on sleep ecology, including modeling predation risk, the ecological roles of REM and NREM sleep, and the effects of habitat heterogeneity and stochasticity.

## Authors' contributions

AA wrote the simulation code (using C++ language and Qt Trolltech ^® ^libraries for the graphical user interface), carried out data analysis, participated in the design of the study, and helped write the manuscript. PM participated in the design of the study and helped in the revision of the manuscript. CLN conceived the study, participated in its design and coordination, and helped write the manuscript. All authors read and approved the final manuscript.

## References

[B1] Ganguly-Fitzgerald I, Donela J, shaw PJ (2006). Waking experience affects sleep need in Drosophila. Science.

[B2] Seymour JE, Carrette TJ, Sutherland PA (2004). Do box jellyfish sleep at night?. Medical Journal of Australia.

[B3] Roth TC, Lesku JA, Amlaner CJ, Lima SL (2006). A phylogenetic analysis of the correlates of sleep in birds. Journal of Sleep Research.

[B4] Nicolau M, Akaarir M, Gamundi A, Gonzalez J, Rial R (2000). Why we sleep: the evolutionary pathway to the mammalian sleep. Progress in Neurobiology.

[B5] Zeppelin H, Siegel GM, Tobler I, Kryger MH, Roth T, Dement WC (2005). Mammalian sleep. Principles and practice of sleep medicine.

[B6] Affanni JM, Cervino CO, Marcos HJA (2001). Absence of penile erections during paradoxical sleep. Peculiar penile events during wakefulness and slow wave sleep in armadillo. Journal of Sleep Research.

[B7] Immelman K, Gebbing H (1962). Schlaf bei Giraffiden. Zeitschrift fur Tierpsychologie.

[B8] Kristal MB, Noonan M (1979). Note on Sleep in Captive Giraffes (*Giraffa camelopardalis Reticulata*). South Africa Journal of Zoology.

[B9] Ball NJ, Stampi C (1992). The phasing of sleep in mammals. Why we nap: Evolution, chronobiology and functions of polyphasic and ultrashort sleep.

[B10] Tobler I, Dinges DF, broughton RJ (1989). Napping and polyphasic sleep in mammals. Sleep and Alertness: Chronobiological, Behavioral Medical Aspects of Napping.

[B11] Elgar MA, Pagel MD, Harvey PH (1988). Sleep in Mammals. Animal Behavior.

[B12] Allison T, Cicchetti DV (1976). Sleep in Mammals – Ecological and Constitutional Correlates. Science.

[B13] Zeppelin H, Rechtschaffen A (1974). Mammalian Sleep, Longevity, and Energy Metabolism. Brain, Behavior and Evolution.

[B14] Maquet P, Smith C, Stickgold R (2003). Sleep and Brain Plasticity.

[B15] McNamara P (2004). An evolutionary psychology of sleep and dreams.

[B16] Tobler I, Kryger MH, Roth T, Dement WC (2005). Phylogeny of sleep regulations. Principles and practice of sleep medicine.

[B17] Churchfield S (1996). Ecology of very small terrestrial mammals. Symp Zool Soc Lond.

[B18] Hansky I, Merritt JF, Kirkland GL, Rose RK (1994). Population biological consequences of body size. Advances in the biology of shrews.

[B19] Lesku JA, Roth TC, Amlaner CJ, Lima SL (2006). A phylogenetical analysis of sleep architecture in mammals: the integration of anatomy, physiology, and ecology. The American Naturalist.

[B20] Lima SL, Rattenborg NC, Lesku JA, Amlaner CJ (2005). Sleeping under the risk of predation. Animal Behavior.

[B21] Capellini I, Barton RA, McNamara P, Preston B, Nunn CL Ecology and Evolution of Mammalian Sleep. Evolution.

[B22] Capellini I, Barton RA, McNamara P, Preston B, Nunn CL Ecology and evolution of the Phasing of Sleep and REM-nREM Sleep Cycle. Functional Ecology.

[B23] Anderson JR (1998). Sleep, sleeping sites, and sleep-related activities: Awakening to their significance. American Journal of Primatology.

[B24] Hansell HM (1993). The Ecological Impact of Animal Nests and Burrows. Functional Ecology.

[B25] Grimm V, Railsback SF (2005). Individual-based Modeling and Ecology.

[B26] Holland JH (1975). Adaptation in Natural and Artificial Systems.

[B27] Mirolli M, Parisi D, Banzhaf W, Christaller T, Dittrich P, Kim JT, Ziegler J (2003). Artificial Organisms that sleep. Advances in Artificial Life – Proceedings of ECAL 2003.

[B28] Beckman BE, McKinley PK, Ofria C, Almeida e Costa F, Rocha LM, Costa E, Harvey I, Coutinho A (2007). Evolution of an Adaptive Sleep Response in Digital Organisms. Advances in Artificial Life – Proceedings of ECAL 2007.

[B29] Lima SL, Rattenborg NC (2007). A behavioural shutdown can make sleeping safer: a strategic perspective in the function of sleep. Animal Behavior.

[B30] Grimm V, Berger U, Bastiansen F, Eliassen S, Ginot V, Giske J, Goss-Custard J, Grand T, Heinz SK, Huse G, Huth A, Jepsen JU, Jørgensen C, Mooij WM, Müller B, Pe'er G, Piou C, Railsback SF, Robbins AM, Robbins MM, Rossmanith E, Rüger N, Strand E, Souissi S, Stillman RA, Vabø R, Visser U, DeAngelis DL (2006). A standard protocol for describing individual-based and agent-based models. Ecological Modelling.

[B31] Little RJA, Rubin DB (1987). Statistical Analysis with Missing Data.

[B32] Alvarenga TAF, Andersen ML, Papale LA, Antuires IB, Tufik S (2005). Influence of long-term food restriction on sleep patterns in male rats. Brain Research.

[B33] Kummer H (1968). Social Organization of Hamadryas: A Field Study.

